# Severe Iron Deficiency Anemia Due to Late Presentation of Congenital Diaphragmatic Hernia in a Toddler

**DOI:** 10.5505/tjh.2012.92609

**Published:** 2012-12-05

**Authors:** Nazan Sarper, Emine Zengin, Suar Çakı Kılıç, Melih Tugay, Ayşen Aydoğan, Özlem Kayabey

**Affiliations:** 1 Kocaeli University, Pediatric Hematology, Kocaeli, Turkey; 2 Kocaeli University, Pediatric Surgery, Kocaeli, Turkey; 3 Kocaeli University, Pediatric Gastroenterology, Kocaeli, Turkey

**To the Editor, **

A 3-year-old female toddler was referred to our pediatric emergency unit with a 2-week history of fatigue, anorexia, progressive pallor, and vomiting. Medical history showed that iron deficiency anemia was diagnosed one year before and oral iron-sulfate was given. She also had a one year history of intermittent vomiting. Her diet seemed adequate in iron-rich foods. Chest X-ray and abdominal ultrasonographic examination performed in a medical center were normal. Complete blood count findings were as follows: Hb: 1.7 g/dL; WBC count: 8.0 x 10^9^/L; ANC: 4.6 x 10^9^/L; Hct: 6.9%; RBC count: 1.19 x 10^12^/L; RDW: 20.5; MCV: 58 fL; Plt count: 485 x 10^9^/L; absolute reticulocyte count: 45.5 x 10^9^/L (normal: 50-150 x 10^9^/L). Peripheral blood smear showed hypochromic microcytic red cells, polychromasia, and nucleated red cells. Serum iron was 5 μg/dL, total iron binding capacity was 450 μg/dL, and ferritin was <1 ng/mL. 

She was hospitalized and given packed red cell transfusion. The following day she suddenly developed respiratory distress after breakfast. Chest X-ray showed a radiolucent lesion in the right paracardiac area (Figure). Congenital diaphragmatic hernia (CDH) was confirmed via barium gastrointestinal X-ray and computed tomography of the chest. During surgery the retrosternal diaphragmatic hernia sack was excised, and the stomach, intestines, and transverse colon were reduced to the abdominal cavity. Nissen fundoplication was also performed in an effort to prevent postoperative gastroesophageal reflux; however, another defect was noted on the left posterolateral side of the diaphragm. The defect was repaired and Thal anterior fundoplication was performed. The patient had both Morgagni and Bochdalek defects 

At 6 weeks post surgery the patient had no gastrointestinal symptoms, but her Hb dropped to 7.9 g/dL and fecal occult blood test findings were positive (Hct: 25.6%; RBC count: 3.63 x 10^12^/L; MCV: 70.5 fL; MCH: 21.9; MCHC: 31; RDW: 24; WBC count: 9.8 x 10^9^/L; ANC: 4.3 x 10^9^/L, Plt count: 453 x 10^9^/L; absolute reticulocyte count: 11 x 10^9^/L) [normal 50-150 x 10^9^/L]). Peripheral blood smear showed hypochromic microcytic red cells. Esophagogastroduodenoscopy and esophageal biopsy showed erosions, gastroesophageal reflux, chronic inflammation, and hyperplasia of the epithelium. A proton pump inhibitor, domperidone, and anti-acid medications were started. Iron sucrose was administered twice weekly for 3 weeks and the Hb increased (Hb: 12.2 g/dL; Htc: 34.6%; RBC count: 4.76 x 10^9^/L; MCV: 72fL; MCH: 25.6; MCHC: 35.1; RDW: 17.4; WBC count: 12.2 x 10^9^/L; ANC: 8.2 x 10^9^/L; Plt count: 348 x 10^9^/L, absolute reticulocyte count: 85 x 10^9^/L [normal: 50-150 x 10^9^/L]; ferritin: 16 ng/mL). Blood smear showed normal red cells. At the 2-year follow-up the patient had no gastrointestinal symptoms or anemia. 

The coexistence of Morgagni and Bochdalek defects is rarely reported [1]. About 5%-30% of CDH cases present after the neonatal period, which poses a diagnostic challenge [2]. Such symptoms as vomiting and respiratory distress may be acute or intermittent—sometimes due to gastric volvulus or spontaneous reduction of the hernia to the abdomen. During asymptomatic periods imaging findings may be normal [3]. The presented patient had normal abdominal ultrasound and chest X-ray findings before referral to our department. The presented patient most probably had chronic occult blood loss from the gastrointestinal tract due to reflux esophagitis and mechanical trauma to the diaphragm. Sinaki et al. reported 2 patients—a 19 month-old and 6-year-old—that presented with gastrointestinal symptoms and an Hb of 5.8 g/dL and 6 g/dL, respectively [4]. Zaki et al. reported a 5-year-old patient with persistent anemia that did not respond to adequate hematinics and blood transfusion. The patient had abdominal pain, melena, an Hb of 4.8 g/dL, and multiple linear gastric erosions on the mucosal folds of the lesser curve of the stomach [5]. 

CDH must be included in the differential diagnosis of severe iron deficiency anemia in the absence of such obvious causes as nutritional deficiency, melena, hematochezia, and malabsorption. Physicians must be aware that a history of intermittent vomiting and/or sudden onset respiratory distress in young children are indications for imaging of the upper gastrointestinal tract and thorax.

## Figures and Tables

**Figure:**
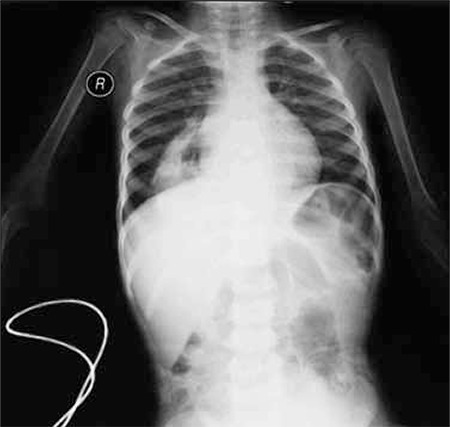
X-ray of the thorax showing the radiolucent area with clear borders in the right paracardiac region.
